# COVID-19 infection and vaccination uptake in men and gender-diverse people who have sex with men in the UK: analyses of a large, online community cross-sectional survey (RiiSH-COVID) undertaken November–December 2021

**DOI:** 10.1186/s12889-023-15779-5

**Published:** 2023-05-05

**Authors:** Dana Ogaz, Hester Allen, David Reid, Jack R. G. Brown, Alison R. Howarth, Caisey V. Pulford, Catherine H. Mercer, John Saunders, Gwenda Hughes, Hamish Mohammed

**Affiliations:** 1 Blood Safety, Hepatitis, STI & HIV Division, UK Health Security Agency, 61 Colindale Avenue, London, NW9 5EQ UK; 2 The National Institute for Health and Care Research Health Protection Research Unit in Blood Borne and Sexually Transmitted Infections at University College London in partnership with the, UK Health Security Agency, London, UK; 3 COVID-19 Vaccines and Epidemiology Division, UK Health Security Agency, London, UK; 4 Institute for Global Health, University College London, London, UK; 5 Sigma Research, Public Health, Environments and Society, London School of Hygiene & Tropical Medicine, London, UK; 6 UK Public Health Rapid Support Team, London School of Hygiene & Tropical Medicine, London, UK

**Keywords:** COVID-19, SARS-CoV-2, Vaccination, Long COVID, Gender-diverse, Men who have sex with men, MSM, Transgender, Nonbinary, Gender minority, Sexual minority, United Kingdom

## Abstract

**Background:**

Men and gender-diverse people who have sex with men are disproportionately affected by health conditions associated with increased risk of severe illness due to COVID-19 infection.

**Methods:**

An online cross-sectional survey of men and gender-diverse people who have sex with men in the UK recruited via social networking and dating applications from 22 November-12 December 2021. Eligible participants included self-identifying men, transgender women, or gender-diverse individuals assigned male at birth (AMAB), aged ≥ 16, who were UK residents, and self-reported having had sex with an individual AMAB in the last year. We calculated self-reported COVID-19 test-positivity, proportion reporting long COVID, and COVID-19 vaccination uptake anytime from pandemic start to survey completion (November/December 2021). Logistic regression was used to assess sociodemographic, clinical, and behavioural characteristics associated with SARS-CoV-2 (COVID-19) test positivity and complete vaccination (≥ 2 vaccine doses).

**Results:**

Among 1,039 participants (88.1% white, median age 41 years [interquartile range: 31-51]), 18.6% (95% CI: 16.3%-21.1%) reported COVID-19 test positivity, 8.3% (95% CI: 6.7%-10.1%) long COVID, and 94.5% (95% CI: 93.3%-96.1%) complete COVID-19 vaccination through late 2021. In multivariable models, COVID-19 test positivity was associated with UK country of residence (aOR: 2.22 [95% CI: 1.26-3.92], England vs outside England) and employment (aOR: 1.55 [95% CI: 1.01-2.38], current employment vs not employed). Complete COVID-19 vaccination was associated with age (aOR: 1.04 [95% CI: 1.01-1.06], per increasing year), gender (aOR: 0.26 [95% CI: 0.09-0.72], gender minority vs cisgender), education (aOR: 2.11 [95% CI: 1.12-3.98], degree-level or higher vs below degree-level), employment (aOR: 2.07 [95% CI: 1.08-3.94], current employment vs not employed), relationship status (aOR: 0.50 [95% CI: 0.25-1.00], single vs in a relationship), COVID-19 infection history (aOR: 0.47 [95% CI: 0.25-0.88], test positivity or self-perceived infection vs no history), known HPV vaccination (aOR: 3.32 [95% CI: 1.43-7.75]), and low self-worth (aOR: 0.29 [95% CI: 0.15-0.54]).

**Conclusions:**

In this community sample, COVID-19 vaccine uptake was high overall, though lower among younger age-groups, gender minorities, and those with poorer well-being. Efforts are needed to limit COVID-19 related exacerbation of health inequalities in groups who already experience a greater burden of poor health relative to other men who have sex with men.

**Supplementary Information:**

The online version contains supplementary material available at 10.1186/s12889-023-15779-5.

## Background

COVID-19 (SARS-CoV-2) was first detected in the United Kingdom (UK) in January 2020. Through December 2021, the number of cases surpassed 13.5 million [1], as the UK experienced multiple COVID-19 waves [2]. Intermittent national lockdowns were put into place from March 2020 with substantial easing of public health measures in July/August 2021.

The national COVID-19 vaccination programme began in December 2020, where roll-out of two-dose vaccination schedules were prioritised for older adults and those with clinical vulnerability, which included people living with HIV (PLWHIV) if immune function was weakened [3]. By June 2021, all adults aged ≥ 18 had access to a first dose, while booster doses (i.e. a third vaccine dose) were available to select priority groups from September 2021, with wider availability to all adults from December 2021 [4].

In England, the age-standardised percentage of double-vaccinated adults peaked at 86.9% from December 2021 through May 2022 (85.4% in men) [5]. Despite high vaccination levels, national surveillance suggests inequalities in vaccination uptake where, among men, vaccination remains highest in those of White British ethnicity (88.0% by May 2022), and lowest in men of Black African (68.3%) and Black Caribbean (56.3%) ethnicities.

To date, increased COVID-19 related clinical severity and mortality in the UK have been described among men, people of Black or Asian ethnicity, and PLWHIV [6–9]. The prevalence of long COVID, described as the continuation or development of new symptoms following initial COVID-19 infection [10] and considered an emerging public health challenge, was estimated in 2% of UK men in January 2022 [11].

As COVID-19 continues to widen health inequalities in the UK [7], it is unclear to what extent COVID-19 has impacted men and gender-diverse people who have sex with men. Despite being disproportionately affected by health conditions associated with increased risk of severe illness due to COVID-19 infection [12, 13], available COVID-related literature for men who have sex with men has primarily focused on changing sexual risk and behaviours following COVID-associated lockdowns.

Given this lack of evidence and the opportunity to examine further intersectional inequalities, we used data from the most recent round of the “Reducing inequalities in Sexual Health (RiiSH)-COVID” survey, part of a series of large, online UK-based community surveys, to assess self-reported COVID-19 test positivity and prevalence of long COVID, as well as self-reported COVID-19 vaccination uptake in men and gender-diverse people who have sex with men.

## Methods

The RiiSH-COVID surveys are a series of four, online, cross-sectional surveys assessing the impact of COVID-19 and related restrictions on health and wellbeing, sexual behaviour, and service-use among a community sample of men and gender-diverse people who have sex with men in the UK. Each round was fielded during different stages of the pandemic, where the fourth round, and data source for this paper, was deployed from 22 November–12 December 2021. Survey methodology and recruitment through social networking and geospatial dating applications have been previously described [14, 15]. Briefly, eligible participants included self-identifying men (cisgender/transgender), transgender women, or gender-diverse individuals assigned male at birth (AMAB), aged ≥ 16, UK residents, who self-reported having had sex with a man (cisgender/transgender) or gender-diverse individual AMAB in the last year. Online consent was obtained from all participants and no incentive was offered to participate.

### Statistical analyses

#### COVID-19 positivity and long COVID history

COVID-19 test positivity was calculated among those who, from pandemic start through survey completion (November/December 2021), ever self-reported having a swab (PCR [virus] or lateral flow test [antigen]) or blood (antibody) test (where an antibody test was prior to vaccination in those vaccinated). Recent positivity was defined as having a positive test from August-December 2021.

Given differential testing availability over the course of the epidemic in the UK, we undertook a sensitivity analysis examining the prevalence of a COVID-19 infection history based on 1) a self-reported prior positive swab or blood test, or 2) a self-perceived COVID-19 infection in those without a prior positive test or testing history (note, 1 and 2 mutually exclusive).

We calculated the prevalence of self-reported long COVID in all participants, irrespective of COVID-19 test history, modelled on UK Office for National Statistics (ONS) estimates of self-reported long COVID in representative national surveys [11, 16].

In the cohort of participants asked whether they had experienced long COVID (those with COVID-19 infection history, i.e., self-reported COVID-19 test positivity or self-perception) (Appendix 1), we examined differences (*p* < 0.05) in sociodemographic, clinical, behaviour and personal well-being characteristics by long COVID self-report using Pearson’s chi-squared and Fisher’s exact tests.

#### COVID-19 vaccine offer and uptake

COVID-19 vaccine offer and uptake, self-reported anytime during pandemic start through survey completion, was assessed in all participants. Vaccine uptake was defined as the percentage of participants reporting ≥ 1 vaccine dose from pandemic start to survey completion. Complete vaccination (hereafter, complete vaccination) was defined as reporting ≥ 2 vaccine doses.

All calculated percentages in COVID-19 positivity, long COVID, and vaccination uptake outcomes are reported with associated 95% confidence intervals (CI) (Clopper-Pearson).

#### Factors associated with COVID-19 test positivity and complete COVID-19 vaccination

Using Pearson’s chi-squared test and binary logistic regression, we used separate models to assess factors associated with COVID-19 test positivity and complete COVID-19 vaccination, respectively. Multivariable models were adjusted for covariates where bivariate association was *p* < 0.10. Evidence of association in multivariable models was considered where *p* < 0.05. Unadjusted and adjusted odds ratios, 95% CIs, and p-values derived from the likelihood ratio test are presented. A sensitivity analysis assessing factors associated with COVID-19 infection history (i.e., test positivity or self-perceived infection) was also carried out.

Covariates used for bivariate and multivariable analyses included sociodemographic characteristics (age-group, ethnicity, gender, sexual orientation, country of birth, UK country of residence, education-level, employment, household composition [living alone or not], relationship status) and clinical history (HIV status, having a medical condition identified as placing someone at greater risk of severe illness from COVID-19 based on national guidelines [hereafter, known COVID-19 shielding] [3]). Mental health and personal well-being indicators derived from the UK ONS ‘Opinions and Lifestyle Survey’, a weekly survey assessing well-being through the COVID period, were integrated into RiiSH-COVID surveys, and used across analyses. Likert-scale responses (11 point) (“Overall, how anxious did you feel yesterday?” and “Overall, to what extent do you feel the things you do in your life are worthwhile”) were dichotomised to create a measure of high anxiety and low self-worth as per ONS standardisation [17, 18].

Additional covariates included: number of male physical sex partners in the preceding 3–4 month lookback period and PrEP use since first lockdown (i.e. after 23 March 2020), considered transmission risk/behavioural confounders in COVID-19 test positivity and vaccination analyses; self-reported COVID-19 infection history (self-reported test positivity or self-perceived infection) and human papillomavirus (HPV) vaccination history (self-report of ≥ 1 HPV vaccine doses) used as clinical history and vaccine acceptance indicators in COVID-19 vaccination analyses.

Covariate selection aimed to limit collinearity where multiple covariates were thought to contribute to similar measures (e.g., transmission risk, mental health and well-being, vaccine acceptance indicators). Age and ethnicity were selected *a priori* for multivariate model inclusion.

Due to limited numbers of participants in gender minority groups (transgender and nonbinary individuals), sexual minority groups (straight and bisexual-identifying individuals), and minority ethnic groups (Black, Asian, other minority ethnic groups), these participants were grouped in analyses.

All analyses were conducted using Stata v.15.0 (StataCorp, College Station, TX, USA).

## Results

Between 22 November-12 December 2021, 1,039 men and gender-diverse people who have sex with men took part in the fourth RiiSH-COVID survey (Appendix 1, 2). Missing data were limited as most responses were compulsory.

Participants had a median age of 41 (interquartile range [IQR] 31–51). Most were of White ethnicity (88.1%), resided in England (85.6%), and reported current employment (75.7%). Nearly all self-identified as cisgender male (95.7%). Known COVID-19 shielding was reported in 17.8% of participants (43.3% [52/120] in PLWHIV); further description of RiiSH-COVID participants is found in Tables 1, 2 and 3.

**Table 1:** Characeristics of, and factors associated with self-reported COVID-19 test positivity among, RiiSH-COVID participants, November–December 2021

Sociodemographic characteristics	% All RiiSH-COVID participants (N)	% COVID-19 test positive^a^ (n/N)		uOR	95% CI		aOR	95% CI
	[column %]	[row %]						
All participants	100.0 (1039)	18.6 (193/1039)						
Median age [IQR]:	41 [33-51]	37 [30-48]						
Age (continuous)^b^	100.0 (1039)			0.98	0.97-1.00		0.99	0.98-1.00
			*p*-value	0.008		*p*-value	0.146	
Age-group								
16–29	21.9 (227)	20.3 (46/227)		1.00	(ref)		…	…
30–44	37.2 (386)	21.0 (81/386)		1.04	0.70-1.57		…	…
45–59	31.2 (324)	17.3 (56/324)		0.82	0.53-1.27		…	…
≥ 60	9.8 (102)	9.8 (10/102)		0.43	0.21-0.89		…	…
			*p*-value	0.041				
Ethnicity^b^								
White	88.1 (915)	18.7 (171/915)		1.00	(ref)		1.00	(ref)
Ethnic minority	11.9 (124)	17.7 (22/124)		0.94	0.58-1.53		0.72	0.43-1.20
			*p*-value	0.798		*p*-value	0.196	
Gender								
Cisgender male	95.7 (994)	18.5 (184/994)		1.00	(ref)		…	…
Gender minority	4.3 (45)	20.0 (9/45)		1.10	0.52-2.32		…	…
			*p*-value	0.803				
Sexual orientation								
Gay/homosexual	80.9 (841)	18.7 (157/841)		1.00	(ref)		…	…
Bisexual or straight	19.1 (198)	18.2 (36/198)		0.97	0.65-1.45		…	…
			*p*-value	0.874				
UK-born								
No	23.7 (246)	19.1 (47/246)		1.00	(ref)		…	…
Yes	76.3 (793)	18.4 (146/793)		0.96	0.66-1.38		…	…
			*p*-value	0.807				
Country of residence in the UK								
Wales, Scotland, or Northern Ireland	14.4 (150)	10.0 (15/150)		1.00	(ref)		1.00	(ref)
England	85.6 (889)	20.0 (178/889)		2.25	1.29-3.94		2.22	1.26-3.92
			*p*-value	0.002		*p*-value	0.003	
Education								
Below degree-level	43.2 (449)	16.0 (72/449)		1.00	(ref)		1.00	(ref)
Degree-level or higher	56.8 (590)	20.5 (121/590)		1.35	0.98-1.86		1.33	0.95-1.87
			*p*-value	0.065		*p*-value	0.090	
Current employment								
No	24.4 (253)	12.7 (32/253)		1.00	(ref)		1.00	(ref)
Yes	75.7 (786)	20.5 (161/786)		1.45	1.09-1.92		1.55	1.01-2.38
			*p*-value	0.011		*p*-value	0.038	
Relationship status								
In a relationship	48.4 (503)	18.7 (94/503)		1.00			…	…
Single	51.6 (536)	18.5 (99/536)		0.99	0.72-1.35		…	…
			*p*-value	0.928				
**Clinical history**								
HIV status								
Negative/unknown	88.5 (919)	19.0 (175/9919)		1.00	(ref)		…	…
Living with HIV	11.6 (120)	15.0 (18/120)		0.75	0.44-1.27		…	…
			*p*-value	0.273				
Known COVID-19 shielding^c^								
No	82.2 (854)	19.7 (168/854)		1.00	(ref)		1.00	(ref)
Yes	17.8 (185)	13.5 (25/185)		0.64	0.41-1.00		0.72	0.45-1.16
			*p*-value	0.044		*p*-value	0.164	
COVID-19 vaccination status								
None/only one dose	5.2 (54)	27.8 (15/54)		2.02	1.05-3.85		2.18	1.07-4.42
Two doses	52.2 (542)	19.7 (107/542)		1.29	0.93-1.79		1.07	0.74-1.56
Two doses and booster	42.6 (443)	16.0 (71/443)		1.00	(ref)		1.00	(ref)
			*p*-value	0.075		*p*-value	0.104	
**Transmission risk indicators**								
Living alone								
No	60.6 (630)	20.6 (130/630)		1.00	(ref)		1.00	(ref)
Yes	39.4 (409)	15.4 (63/409)		0.70	0.50-0.97		0.77	0.54-1.08
			*p*-value	0.033		*p*-value	0.129	
Male physical sex partners in preceding 3–4 months								
No partners	8.2 (85)	16.5 (14/85)		0.84	0.45-1.56		…	…
1 partner	20.4 (212)	16.5 (35/212)		0.84	0.54-1.30		…	…
2–4 partners	29.5 (306)	19.9 (61/306)		1.06	0.73-1.53		…	…
≥ 5 partners	42.0 (436)	19.0 (83/436)		1.00	(ref)		…	…
			*p*-value	0.817				
PrEP history since first lockdown								
No known PrEP use^d^	69.8 (725)	17.1 (124/725)		1.00	(ref)		1.00	(ref)
PrEP use	30.2 (314)	22.0 (69/314)		1.37	0.98-1.90		1.27	0.90-1.79
			*p*-value	0.067		*p*-value	0.176	
**Mental health and personal well-being indicators**								
High anxiety								
No	64.0 (660)	19.4 (128/660)		1.00	(ref)		…	…
Yes	36.1 (372)	16.9 (63/372)		0.85	0.61-1.18		…	…
			*p*-value	0.326				
Low self-worth								
No	83.4 (861)	18.8 (162/861)		1.00	(ref)		…	…
Yes	16.6 (171)	17.0 (29/171)		0.88	0.57-1.36		…	…
			*p*-value	0.565				

^a^Participants with self-reported COVID-19 test positivity. Those without COVID-19 test positivity include those without a known positive test (*n* = 793) and those without a test history (*n* = 53). ^b^Specified *a priori* for model inclusion. ^c^Self-report of a medical condition identified as placing someone at greater risk of severe illness from COVID-19 as described by national advice. ^d^Includes persons living with HIV (*n* = 120) where 15.0% (18/120) had test positivity. uOR = unadjusted odds ratio. aOR = adjusted odds ratio. *95% CI* 95% confidence interval. Likelihood ratio test (LRT) *p*-values. *IQR* Interquartile range. Gender minority = trans man, trans woman, or gender-diverse person. All observations included in adjusted model (*N* = 1039). Missing values: High anxiety (*n* = 7), low self-worth (*n* = 7)

### COVID-19 positivity and long COVID

Most participants (95.0%) reported having had at least one COVID-19 test from pandemic start through survey completion, where 18.6% (95% CI: 16.3%-21.1%) reported test positivity (median age 37 [IQR: 30–48]) (Table 1, Appendix 1). Nearly half of those reporting a positive test (41.5% [80/193]) reported recent positivity (7.7% of all participants). In our sensitivity analysis, 32.9% (95% CI: 30.0%-35.8%) of all participants reported a COVID-19 infection history (Appendix 1).

Long COVID was self-reported in 8.3% (95% CI: 6.7%-10.1%, *n* = 86) of all participants. Among the subgroup of participants reporting a COVID-19 infection history (*n* = 341), those self-reporting long COVID (median age 40 [IQR: 32–53]) were more likely to report prior hospitalisation due to COVID symptoms (8.1% [7/86] vs 2.8% [7/255], *p* = 0.029) and educational qualifications below degree-level (48.8% [42/86] vs 35.7% [91/255], *p* = 0.031) relative to those without self-reported long COVID. There were no differences in complete vaccination by self-reported long COVID (90.7% [78/86] vs 92.9% [237/255], p = 0.498) (Table 2).

**Table 2:** Characeristics of, and factors associated with self-reporting long COVID among RiiSH-COVID participants, November–December 2021

		Long COVID self-report		
**Sociodemographic characteristics**	% All RiiSH-COVID participants with a COVID-19 infection history^a^ (N)	% Yes (n)	% No (n)	*p*-value^b^
	[column %]	[column %]	[column %]	
All participants	100.0 (341)	100.0 (86)	100.0 (255)	
Median age [IQR]	39 [30-49]	40 [32-53]	38 [30-48]	
Age-group				
16–29	23.2 (79)	19.8 (17)	24.3 (62)	
30–44	40.2 (137)	39.5 (34)	40.4 (103)	
45–59	30.5 (104)	31.4 (27)	30.2 (77)	
≥ 60	6.2 (21)	9.3 (8)	5.1 (13)	0.482
Ethnicity				
White	88.9 (303)	91.9 (79)	87.8 (224)	
Ethnic minority	11.1 (38)	8.1 (7)	12.2 (31)	0.306
Gender				
Cisgender male	95.6 (326)	98.8 (85)	94.5 (241)	
Gender minority	4.40 (15)	1.2 (1)	5.5 (14)	0.128^c^
Sexual orientation				
Gay/homosexual	79.2 (270)	80.2 (69)	78.8 (201)	
Bisexual or straight	20.8 (71)	19.8 (17)	21.2 (54)	0.781
UK-born				
No	23.5 (80)	23.3 (20)	23.5 (60)	
Yes	76.5 (261)	76.7 (66)	76.5 (195)	0.959
Residence				
Wales, Scotland, or Northern Ireland	11.4 (39)	15.1 (13)	10.2 (26)	
England	88.6 (302)	84.9 (73)	89.8 (229)	0.215
Education				
Below degree-level	39.0 (133)	48.8 (42)	35.7 (91)	
Degree-level or higher	61.0 (208)	51.2 (44)	64.3 (164)	0.031
Current employment				
No	19.7 (67)	26.7 (23)	17.3 (44)	
Yes	80.4 (274)	73.3 (63)	82.8 (211)	0.055
Living alone				
No	64.2 (219)	55.8 (48)	67.1 (171)	
Yes	35.8 (122)	44.2 (38)	32.9 (84)	0.060
**Clinical history**				
HIV status				
Negative/unknown	89.7 (306)	90.7 (78)	89.4 (228)	
Living with HIV	10.3 (35)	9.3 (8)	10.6 (27)	0.734
Known COVID-19 shielding^d^				
No	85.9 (293)	82.6 (71)	87.1 (222)	
Yes	14.1 (48)	17.4 (15)	12.9 (33)	0.299
COVID-19 vaccination status				
None/only one dose	7.6 (26)	9.3 (8)	7.1 (18)	
Two or more doses	92.4 (315)	90.7 (78)	92.9 (237)	0.498
Hospitalisation due to COVID symptoms				
No	95.9 (327)	91.9 (79)	97.3 (248)	
Yes	4.10 (14)	8.1 (7)	2.8 (7)	0.029
**Mental health and personal well-being indicators**				
High anxiety				
No	65.6 (221)	57.1 (48)	68.4 (173)	
Yes	34.2 (116)	42.9 (36)	31.6 (80)	0.060
Low self-worth				
No	81.6 (274)	77.4 (65)	82.9 (209)	
Yes	18.5 (62)	22.6 (19)	17.1 (43)	0.256

^a^Participants with self-reported COVID-19 test positivity (*n* = 193) or self-perceived infection (*n* = 148). ^b^Chi-squared *p*-value unless otherwise specified. ^c^Fisher's exact test *p*-value. ^d^Self-report of a medical condition identified as placing someone at greater risk of severe illness from COVID-19 as described by national advice. Gender minority = trans man, trans woman, or gender-diverse person. Missing values: High anxiety (*n* = 4), low self-worth (*n* = 5)

### COVID-19 vaccine offer and uptake

Nearly all participants received a vaccine offer (98.2% [1020/1039]). Complete vaccination was reported by 985 participants (94.8% [95% CI: 93.3%-96.1%]) (Table 3, Appendix 2). Nearly half of all participants reported having had a booster (42.6% [443/1039]; 70.8% [85/120] in PLWHIV).

**Table 3:** Characteristics of, and factors associated with self-reported COVID-19 vaccination history, among RiiSH-COVID participants, November–December 2021

Sociodemographic characteristics	% All RiiSH-COVID participants (N)	% Complete COVID-19 vaccination^a^ (n/N)		uOR	95% CI		aOR	95% CI
	[column %]	[row %]						
All participants	100.0 (1039)	94.8 (985/1039)						
Median age [IQR]:	41 [31-51]	41 [31-52]						
Age (continuous)^b^	100.0 (1039)			1.04	1.02-1.07		1.04	1.01-1.06
			*p*-value	< 0.001		*p*-value	0.003	
Age-group								
16–29	21.9 (227)	90.3 (205/227)		1.00	(ref)		…	…
30–44	37.2 (386)	95.1 (367/386)		3.05	1.45-6.43		…	…
45–59	31.2 (324)	96.6 (313/324)		3.41	1.68-6.91		…	…
≥ 60	9.8 (102)	98.0 (100/102)		5.37	1.24-23.3			
			*p*-value	0.005				
Ethnicity^b^								
White	88.1 (915)	95.3 (872/915)		1.00	(ref)		1.00	(ref)
Ethnic minority	11.9 (124)	91.1 (113/124)		0.51	0.25-1.01		0.55	0.25-1.23
			*p*-value	0.069		*p*-value	0.158	
Gender								
Cisgender male	95.7 (994)	96.5 (950/994)		1.00	(ref)		1.00	(ref)
Gender minority	4.3 (45)	77.8 (35/45)		0.16	0.08-0.35		0.26	0.09-0.72
			*p*-value	< 0.001		*p*-value	0.015	
Sexual orientation								
Gay/homosexual	80.9 (841)	96.0 (807/841)		1.00	(ref)		1.00	(ref)
Bisexual or straight	19.1 (198)	89.9 (178/198)		0.37	0.21-0.67		0.60	0.31-1.18
			*p*-value	0.002		*p*-value	0.149	
UK-born								
No	23.7 (246)	95.9 (236/246)		1.00	(ref)		…	…
Yes	76.3 (793)	94.5 (749/793)		0.72	0.36-1.46		…	…
			*p*-value	0.347				
Country of residence								
Wales, Scotland, Northern Ireland	14.5 (150)	92.7 (139/150)		1.00	(ref)		…	…
England	85.6 (889)	95.2 (846/889)		1.56	0.78-3.09		…	…
			*p*-value	0.224				
Education								
Below degree-level	43.3 (449)	92.2 (414/449)		1.00	(ref)		1.00	(ref)
Degree-level or higher	56.8 (590)	96.8 (571/590)		2.54	1.43-4.50		2.11	1.12-3.98
			*p*-value	0.001		*p*-value	0.019	
Current employment								
No	24.4 (253)	90.1 (228/253)		1.00	(ref)		1.00	(ref)
Yes	75.7 (786)	96.3 (757/786)		2.86	1.64-4.99		2.07	1.08-3.94
			*p*-value	< 0.001		*p*-value	0.030	
Living alone								
No	60.6 (630)	94.9 (598/630)		1.00	(ref)		…	…
Yes	39.4 (409)	94.6 (387/409)		0.94	0.54-1.64		…	…
			*p*-value	0.832				
Relationship status								
In a relationship	48.4 (503)	97.2 (489/503)		1.00	(ref)		1.00	(ref)
Single	51.6 (536)	92.5 (496/536)		0.36	0.19-0.66		0.50	0.25-1.00
			*p*-value	0.001		*p*-value	0.043	
**Clinical history**								
HIV status								
Negative/unknown	88.5 (919)	94.8 (871/919)		1.00	(ref)		…	…
Living with HIV	11.6 (120)	95.0 (114/120)		1.05	0.44-2.50		…	…
			*p*-value	0.917				
Known COVID-19 shielding								
No	82.2 (854)	94.3 (805/854)		1.00	(ref)		1.00	(ref)
Yes	17.8 (185)	97.3 (180/185)		2.19	0.86-5.58		2.26	0.83-6.12
			*p*-value	0.069		*p*-value	0.084	
COVID-19 infection history^c^								
No	67.2 (697)	96.0 (669/697)		1.00	(ref)		1.00	(ref)
Yes	32.9 (341)	92.4 (315/341)		0.51	0.29-0.88		0.47	0.25-0.88
			*p*-value	0.017		*p*-value	0.018	
Known HPV vaccination history								
No	69.0 (717)	93.6 (671/717)		1.00	(ref)		1.00	(ref)
Yes	31.0 (322)	97.5 (314/322)		2.69	1.26-5.77		3.32	1.43-7.75
			*p*-value	0.005		*p*-value	0.002	
**Behavioural indicators**								
Male physical sex partners in preceding 3 months								
No partners	8.2 (85)	90.6 (77/85)		1.00	(ref)		…	…
1 partners	20.4 (212)	96.2 (204/212)		2.65	0.96-7.31		…	…
≥ 2 partners	71.4 (742)	94.9 (704/742)		1.92	0.87-4.27		…	…
			*p*-value	0.177				
**Mental health and personal well-being indicators**								
High anxiety								
No	64.0 (660)	95.0 (627/660)		1.00	(ref)		…	…
Yes	36.1 (372)	94.9 (353/372)		0.98	0.55-1.75		…	…
			*p*-value	0.940				
Low self-worth								
No	83.4 (861)	96.6 (832/861)		1.00	(ref)		1.00	(ref)
Yes	16.6 (171)	86.6 (148/171)		0.22	0.13-0.40		0.29	0.15-0.54
			*p*-value	< 0.001		*p*-value	< 0.001	

^a^Participants self-reporting ≥ 2 COVID-19 vaccine doses through survey completion. Those without complete COVID-19 vaccination include: 15 with uptake of only 1 dose, 32 with no vaccine uptake, and 7 who responded didn't know/prefer not to say. ^b^Specified* a priori* for model inclusion. ^c^Includes those who self-reported COVID-19 test positivity or self-perceived infection. uOR = unadjusted odds ratio. aOR = adjusted odds ratio. 95% CI = 95% confidence interval. Likelihood ratio test (LRT) *p*-values. *IQR* Interquartile range. Gender minority = trans man, trans woman, or gender-diverse person. 1032 observations included in adjusted model. Missing values: High anxiety (*n* = 7), low self-worth (*n* = 7)

### Factors associated with COVID-19 infection and complete COVID-19 vaccination

Bivariate and multivariable associations with COVID-19 test positivity and complete vaccination are found in Tables 1, 3. For both outcomes, we found no bivariate association by HIV status or country of birth.

Following adjustment in multivariable models, evidence of an association with COVID-19 test positivity was found by UK country of residence (aOR: 2.22 [95% CI: 1.26–3.92], England vs outside England) and employment (aOR: 1.55 [95% CI: 1.01–2.38], current employment vs not employed). There was no evidence of independent association to COVID-19 test positivity by age, ethnicity, or COVID-19 vaccination, though those reporting incomplete vaccination (0–1 doses) were twice as likely to report COVID-19 test positivity relative to those having received 3 vaccine doses (aOR: 2.18 [95% CI: 1.07–4.42]).

The likelihood of complete COVID-19 vaccination increased with age (aOR: 1.04 [95% CI: 1.01–1.06], per increasing year), degree-level or higher educational qualifications (aOR: 2.11 [95% CI: 1.12–3.98], vs below degree-level), current employment (aOR: 2.07 [95% CI: 1.08–3.94], vs not employed), and having a known HPV vaccination (aOR: 3.32 [95% CI: 1.43–7.75]) in adjusted multivariable analysis. Lower likelihood of complete vaccination was seen for gender minority groups (aOR: 0.26 [95% CI: 0.09–0.72], vs cisgender), those with a single relationship status (aOR: 0.50 [95% CI: 0.25–1.00], vs in a relationship), a COVID-19 infection history (aOR: 0.47 [95% CI: 0.25–0.88], vs no history), and the report of low self-worth (aOR: 0.29 [95% CI: 0.15–0.54]).

## Discussion

In this large, community sample of men and gender-diverse people who have sex with men, a fifth of participants self-reported testing positive for COVID-19 over the 18 months from pandemic start through late 2021. Across this period, nearly one in ten participants self-reported experiencing long COVID. Complete COVID-19 vaccination was near ubiquitous, as most reported vaccine offer and uptake.

To date, these are the only examinations of COVID-19 positivity outcomes and COVID-19 vaccination uptake among men and gender-diverse people who have sex with men in the UK, important populations who are disproportionately affected by poor health outcomes. While extensive, there was no disaggregate data by gender identity or sexual orientation for our study populations found in UK COVID-19 surveillance outputs. We found few descriptions of COVID-19 outcomes in gender and sexual minorities [19, 20], despite concerted acknowledgement in recognising gender identity as a social health determinant and the need for improved visibility of gender minorities in research and health-reporting [21, 22]. Our study population is broadly representative of prior RiiSH-COVID survey rounds, where fluctuating levels of sexual risk behaviour through the pandemic were reported [15]. Though not wholly comparable, the proportion of participants who self-reported long COVID in this study exceeded the most recently available self-reported estimates in UK men in July 2022 (< 3%) [16].

In our study, those who had ever tested positive for COVID-19 were more likely to live in England and report current employment. Our results likely reflect higher absolute COVID-19 case numbers in England [1], though test positivity varied in the UK through the pandemic [23], and increased case ascertainment facilitated through greater testing accessibility as the pandemic progressed [1]. Higher transmission risk due to greater social mixing, increased in-person working and workplace associated testing as lockdowns had eased, may underpin association to employment. We have no insight as to our survey respondents’ employment type (e.g. public-facing vs working from home), which has been described as a key driver to infection inequalities [7]. To limit misclassification in COVID-19 outcomes, COVID-19 test positivity was utilised in multivariable models given wider circulating respiratory pathogens following lifting of lockdown restrictions [24]. Results from sensitivity analyses incorporating self-perceived infection, show similar direction and magnitude of age and employment related effect estimates, where those with higher educational qualifications and ≥ 2 vaccine doses also had a higher likelihood of an infection history (Appendix 3).

On examining long COVID among the subgroup of participants with a COVID-19 infection history, a higher proportion of those with long COVID self-report, compared to those without, had a prior hospitalisation due to COVID symptoms which may be indicative of infection severity. We found no differences in long COVID by vaccination status, where a recent systematic review suggests reduced risk of long COVID in those vaccinated prior to COVID-19 infection [25]. Given our study design we cannot establish whether reported vaccination preceded infection, a key limitation to interpretation. Unlike a UK-based cohort study of non-hospitalised adults with COVID-19 [26], we found no association between long COVID and age or ethnicity but did find similar socioeconomic inequalities, as those reporting long COVID were less likely to be degree-educated.

Encouragingly, COVID-19 vaccination uptake across participants was high and surpassed population-level estimates of UK men. While our study sample was comprised of younger men relative to the national population [27], complete vaccination exceeded 90% across all age groups. Despite high engagement, age and socioeconomic-related vaccination inequalities were consistent to those nationally reported [5, 28], where participants who were younger, unemployed, and with lower educational qualifications were less likely to report full vaccination despite widespread availability at the time, again suggesting lower levels of deprivation and high health literacy in our sample. We found similar results to an Australian cross-sectional study of gay and bisexual men [29], where COVID-19 vaccination was independently associated with older age and university education in the 28% (358/1280) reporting at least partial vaccination. Unlike this study, however, we found no association to complete COVID-19 vaccination by HIV status, which may be due to differences in vaccination implementation progress in respective study countries. Though age-related uptake differences may reflect the staggered roll-out prioritising older adults, age inequalities have persisted in national surveillance over time following our study period [5].

Our study suggested that HIV status was not associated with COVID-19 test positivity, long COVID, or COVID-19 vaccination in our study population, however, these findings are limited by the relatively small sample size of PLWHIV. Other studies have shown higher likelihood of adverse COVID-related outcomes in PLWHIV [9, 30]. COVID-related restrictions throughout the pandemic may have also affected accessibility to care [31]. In the UK, PLWHIV were considered a priority group for COVID-19 vaccination and a population recommended to shield if immune function was compromised [3]. Among RiiSH-COVID participants living with HIV, we saw a higher proportion of reporting COVID-19 booster vaccine doses, relative to those not known to be HIV positive during the early stages of booster roll-out in the UK. Nearly half of PLWHIV reported shielding, which could have overestimated self-reported shielding in our study sample given high viral suppression noted in the UK [32]; however other co-morbidities experienced by PLWHIV may have contributed to shielding self-report [33].

Similarly, known COVID-19 shielding was not found to be associated with COVID-19 vaccination uptake, COVID-19 test positivity or long COVID outcomes. A national shielding programme was introduced in the UK at the start of the pandemic [3]. We have limited insights to shielding reported in men and gender-diverse people who have sex with men, but levels in our study (17.8%) were similar to those reported in a nationally representative sample of English general practice survey respondents in 2021 (16.9%) [34]. While recommended to minimise infection risk in the extremely clinical vulnerable, studies suggest shielding may have amplified feelings of isolation and contributed to negative effects on mental and physical health [35, 36]. The impact of shielding in men and other gender-diverse individuals should be examined given the risk of compounding mental health inequalities already reported in these groups [37, 38]. Despite their relatively small numbers, further intersectional uptake inequalities, not previously described, were seen in gender minority groups, who also reported the lowest levels of complete vaccination (77.8%). Studies suggest common features of COVID-19 vaccination hesitancy among sexual and gender minority groups that include prior medical trauma, medical mistrust, fear of violence, stigma, and discrimination [39, 40].

Throughout the pandemic, national COVID-19 vaccination efforts sought to minimise structural barriers to uptake, signalling the success of an active, universal vaccination offer with continued accessibility. This contrasts with targeted, often stigma-associated vaccination drives directed to men who have sex with men, where infrequent offer by healthcare staff and infection-associated and population-based stigma have been described as barriers to vaccine uptake [41]. Though our analyses suggest a higher likelihood of COVID-19 vaccination in those reporting HPV vaccination, this should not discount the high levels of complete vaccination in those without. The components underpinning the UK’s COVID-19 vaccination programme, including political will, public buy-in and widespread accessibility, may provide some insights to vaccination efforts for emerging health threats such as mpox [42, 43].

Lastly, we found relationships between mental health and well-being to COVID-19 outcomes. Those reporting low self-worth were less likely to report complete COVID-19 vaccination. While we cannot establish directionality, it is plausible that underlying poor mental health and well-being may have affected vaccination uptake as seen in influenza vaccination studies [44]. However, improvements to mental distress have been reported in adults receiving a first vaccine dose [45]. In longitudinal European analyses, restrictive pandemic policies were associated with poorer mental health [46]. Men who have sex with men have been negatively impacted by the pandemic and related restrictions, with higher levels of anxiety and loneliness reported in those practicing physical distancing, and exacerbated economic, health, and service-use related vulnerabilities [47, 48]. There are similar impacts in transgender and nonbinary individuals as cross-sectional studies suggest disrupted access to gender-affirming care and economic instability brought on by pandemic restrictions has driven poor mental health outcomes [49–51]. While qualitative work in the UK [52] indicates that living with a partner a protective factor of well-being, we found no association to COVID-19 vaccination by household composition but see a lower likelihood of vaccination in those reporting a single relationship status. Where possible, longitudinal exploration of upstream and downstream mental health indicators relative to severe COVID-19 outcomes should be explored considering known mental health inequalities among men and gender-diverse people who have sex with men [37] and in absence of vaccine hesitancy guidance for those with mental health difficulties [53].

### Limitations

This study adds to the limited data characterising the COVID-19 experience among men and gender-diverse people who have sex with men in the UK, however, there are several limitations. Due to its cross-sectional design, we cannot establish the temporality between survey responses and outcomes and have limited information on the timing of COVID-19 positivity, or potential re-infection, and COVID-19 vaccination, as the recall period for COVID-related outcomes spans the pandemic. Because of this, we are unable to assess the impact of vaccination on COVID-19 infections in our study sample. Responses were self-reported and subject to recall and social desirability bias, though we expect anonymity to have limited this. COVID-19 test positivity could be reflective of symptomatic cases as testing availability and COVID-19 presentation evolved through the pandemic and sensitivity analyses indicate high levels of self-perceived infection (though this is also subject to misclassification given the rapid resurgence of other respiratory pathogens as restrictions eased in the UK). While long COVID outcomes exceeded national estimates, results must be interpreted with caution given the likelihood of differences arising from sampling bias. However, given the disproportionate burden of health conditions associated with the increased risk of severe COVID-19 reported in men who have sex with men, results are plausible, but warrant further examination. Though RiiSH-COVID sought to recruit from across the community in contrast to recruiting from sexual health clinics, it nonetheless may not be generalisable to the wider UK populations of men and gender-diverse people who have sex with men (of which we already have limited insight). For all COVID-19 outcomes, further disaggregate, population-level data by gender (beyond binary norms) and sexual orientation were not available through national COVID-19 surveillance outputs, limiting more refined comparisons.

Survey recruitment was exclusively online to allow rapid data collection across the UK. As such, participants may not include those without internet, though most adults (94%) were estimated to have home-based access in 2021 [54] and cross-sectional work has highlighted the use of online social media for socialisation in men who have sex with men adhering to physical distancing restrictions [47]. Our sample may represent those who may have been more socially active through the recent pandemic, as over two-thirds reported ≥ 2 male physical sex partners in the preceding lookback period. However, this could also be attributable to changes in sexual behaviour following vaccination [55], though studies also suggest higher sexual risk in geospatial dating app users [56].

We found no evidence of age-related inequalities in COVID test positivity and long COVID history, however, further studies assessing intersectional inequalities are needed given variation in COVID-related morbidity and mortality reported in the general population. Analyses in minority ethnic groups, and gender and sexual minorities are limited due to small sample sizes and disaggregate analyses in multivariable models were not possible. We acknowledge the differing health needs and COVID-19 experience among these groups but have included estimates for transparency and future review though they may not be representative of wider groups. However, a strength of this study is the rich insights that RiiSH-COVID provides for these key populations not readily represented in national COVID-19 data outputs. This bolsters the need for regular and enhanced surveys of the community, in parallel to national surveillance, to ensure, equitable, and if needed, targeted pandemic responses.

## Conclusions

Findings illustrate high engagement with COVID-19 prevention efforts in our large, community-based sample of men and gender-diverse people who have sex with men, where universal offer and sustained vaccine availability through the pandemic should inform efforts for prevention planning due to the risk of emerging health threats. In the context of mpox prevention, investment in vaccine and clinical services are crucial to reach key populations in need [57]. This study adds insight to the reach and success of the UK’s COVID-19 vaccination programme and a glimpse of COVID-19 infection and long COVID outcomes reported in groups experiencing disproportionate health inequalities. Data collected from RiiSH-COVID are an integral complement to available COVID-19 surveillance.

As the pandemic continues, a systems-level approach should continue to address COVID-19 related knowledge gaps, including vaccine hesitancy, in those engaging with health services and outreach as accessibility rebounds following lifted lockdown restrictions. Among gender minorities, efforts are needed to limit COVID-related exacerbation of health inequalities in those already experiencing greater burden of poor health and service-related stigma relative to other men who have sex with men. Tailored and targeted interventions may be needed to close COVID-19 vaccination uptake gaps to prevent the widening of existing health inequalities due to COVID-19.

Further exploration of COVID-related health outcomes, including long COVID, in men and gender-diverse people who have sex with men are required to identify continued COVID-related impacts and emergence of related health inequalities. The lack of data on these populations in national COVID-19 surveillance and inability to meaningfully benchmark comparisons highlight the importance of integrating sexual orientation and gender identity, where possible, to ensure equitable monitoring and response in the face of new and continuing public health threats.

## Supplementary Information

**Additional file 1.** 

**Additional file 2.** 

**Additional file 3.** 

**Additional file 4.** 

## Data Availability

The data that support the findings of this study are available from University College London (UCL) on reasonable request and with permission from the UK Health Security Agency (UKHSA). Requests can be directed to Dr Hamish Mohammed (hamish.mohammed@ukhsa.gov.uk).

## Abbreviations

AMAB: Assigned male at birth
COVID-19: Coronavirus Disease 2019
ONS: Office for National Statistics
PrEP: HIV pre-exposure prophylaxis
RiiSH-COVID: ‘Reducing inequalities in Sexual Health-COVID’ (survey)
SARS-CoV-2: Severe Acute Respiratory Syndrome Coronavirus 2
UK: United Kingdom
