# Supplementary figures and images for: COVID-19 infection and vaccination uptake in men and gender-diverse people who have sex with men in the UK: analyses of a large, online community cross-sectional survey (RiiSH-COVID) undertaken November–December 2021
Source: BMC Public Health. 2023 May 5;23:829. doi: 10.1186/s12889-023-15779-5 (PMC10161154; doi:10.1186/s12889-023-15779-5)

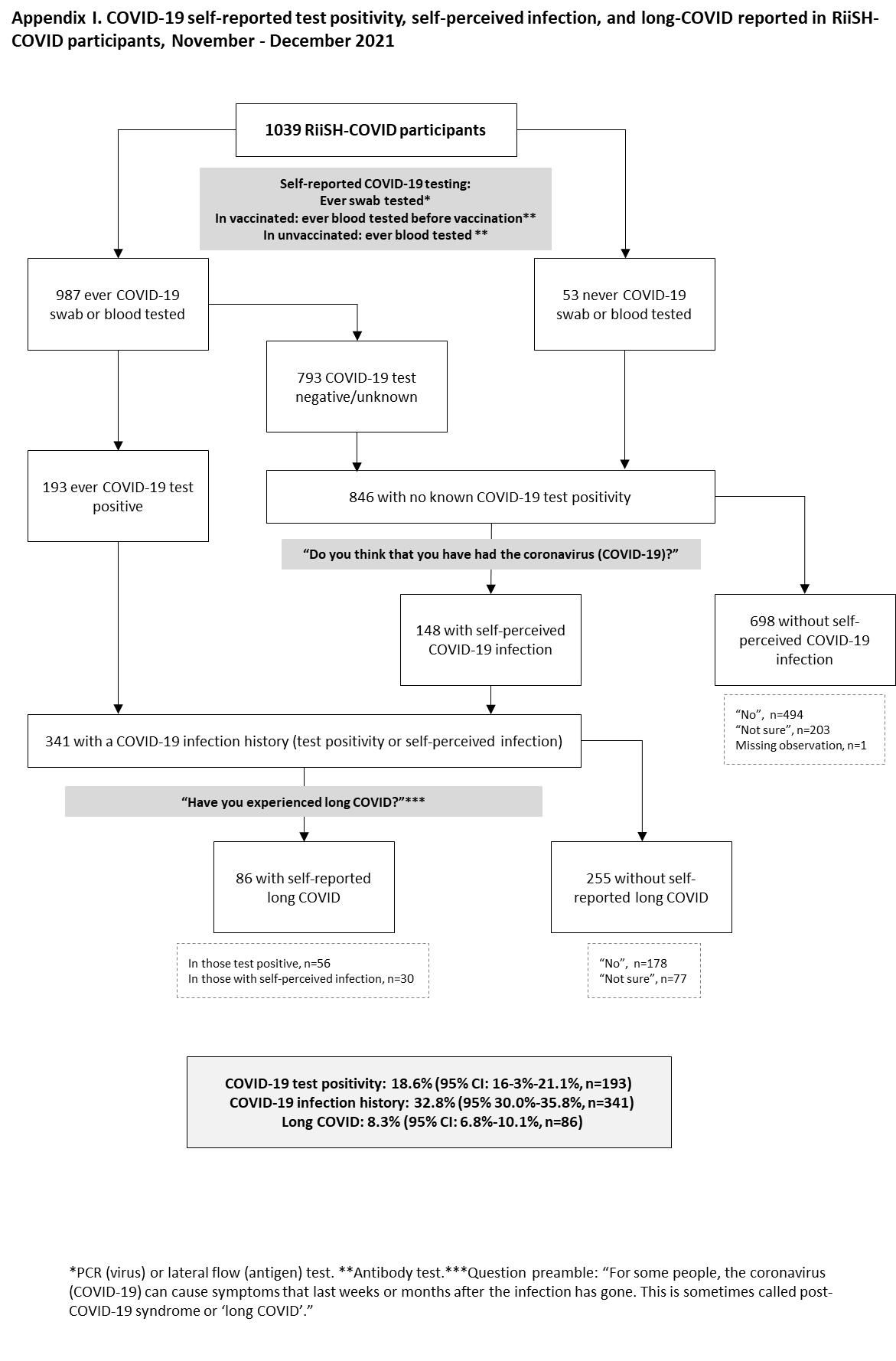

Supplement: Supplementary file 1 — Additional file 1. [file 12889_2023_15779_MOESM1_ESM.docx]

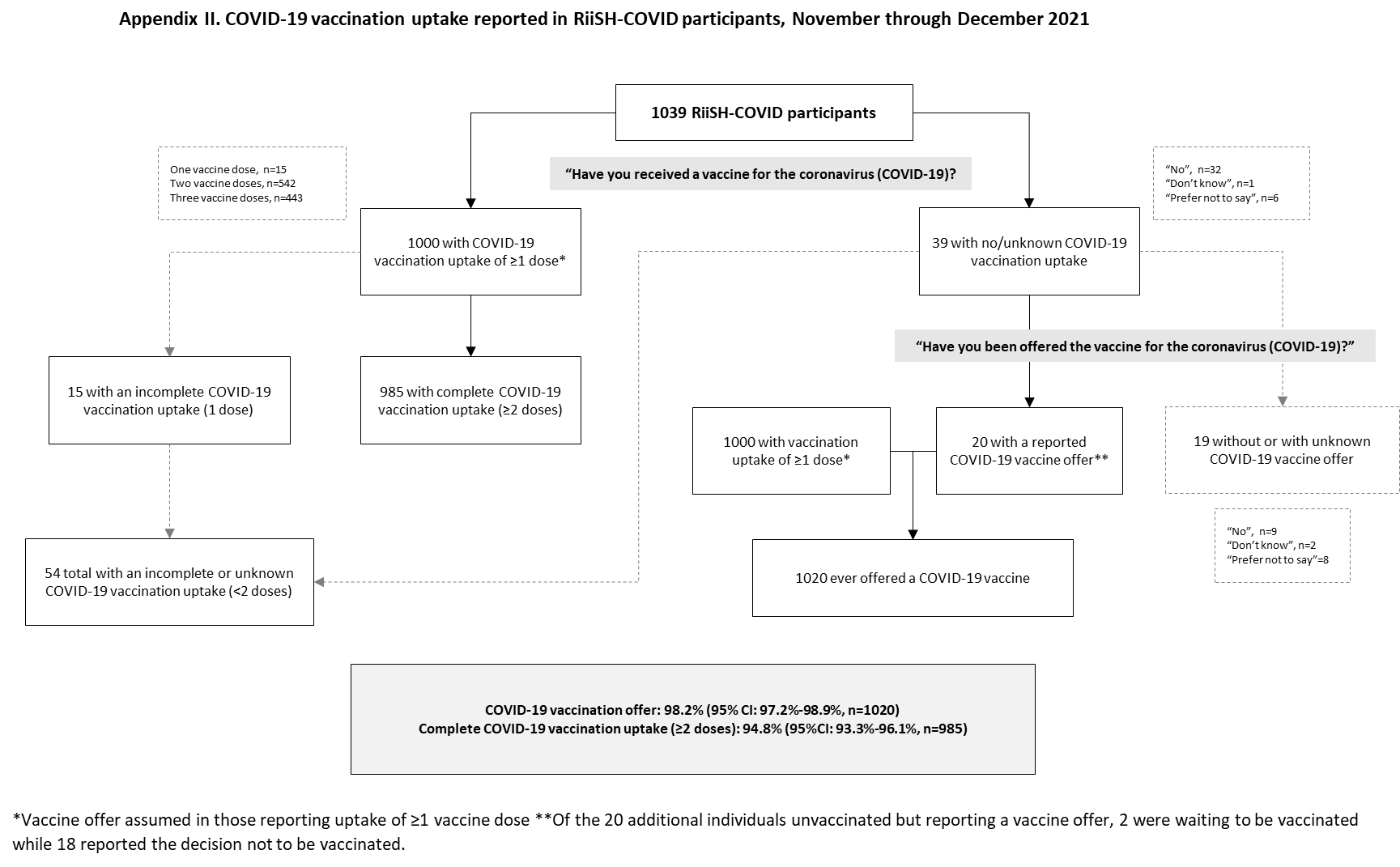

Supplement: Supplementary file 2 — Additional file 2. [file 12889_2023_15779_MOESM2_ESM.docx]
