# Supplementary material for: COVID-19 infection and vaccination uptake in men and gender-diverse people who have sex with men in the UK: analyses of a large, online community cross-sectional survey (RiiSH-COVID) undertaken November–December 2021
Source: BMC Public Health. 2023 May 5;23:829. doi: 10.1186/s12889-023-15779-5 (PMC10161154; doi:10.1186/s12889-023-15779-5)
Supplement: Supplementary file 3 — Additional file 3. [file 12889_2023_15779_MOESM3_ESM.docx]

**Appendix III. Characteristics of, and factors associated with self-reported COVID-19 infection history among RiiSH-COVID participants, November-December 2021**

***continued on following page**

*Participants with self-reported Covid-19 infection history includes those reporting a prior positive test or self-perceived infection (among those with a prior negative test [n=793] or no testing history [n=52]). **Participants providing testing information and self-perceived infection information if had prior negative test or no testing history (1 missing observation). ***Specified a priori for model inclusion. ****Self-report of a medical condition identified as placing someone at greater risk of severe illness from COVID-19 as described by national advice. *****Includes persons living with HIV (n=119) where 29.4% (35/119) had a Covid history. uOR=unadjusted odds ratio. aOR=adjusted odds ratio. 95% CI=95% confidence interval. Likelihood ratio test (LRT) p-values. IQR=Interquartile range. Gender minority=trans man, trans woman, or gender-diverse person. 1031 observations included in adjusted model (N=1038). Missing values: High anxiety (n=6), low self-worth (n=6).
